# The assessment and aetiology of drug-induced ischaemic priapism

**DOI:** 10.1038/s41443-024-01006-1

**Published:** 2024-12-09

**Authors:** Divyen Moodley, Anja Badenhorst, Yahya Choonara, Ahmed Adam

**Affiliations:** 1 Department of Urology, Charlotte Maxeke Johannesburg Academic Hospital, Johannesburg, South Africa; 2 Chair and Head of Pharmacy and Pharmacology, South African DSI-NRF, Research Chair of Pharmaceutical Biomaterials, Drug Delivery and Nanomedicine, Johannesburg, South Africa; 3 Associate Professor in the Division of Urology, Department of Surgery, School of Clinical Medicine, Faculty of Health Sciences, University of the Witwatersrand, Johannesburg, South Africa

**Keywords:** Molecular biology, Drug delivery, Sexual dysfunction

## Abstract

Ischaemic priapism is a urological emergency characterised by a prolonged, painful erection unrelated to sexual stimulation. While several aetiological factors contribute to this condition, the pharmacological causes have gained significant attention in recent years. This narrative review aims to comprehensively assess ischaemic priapism, specifically focusing on its pharmacological aetiology. We propose an approach and assessment strategy to the numerous factors associated with pharmacologically induced ischaemic priapism. By enhancing our understanding of the pharmacological causes of this condition, healthcare professionals can improve patient management and reduce the long-term complications associated with ischaemic priapism.

## Introduction

Ischaemic priapism is a urological emergency characterised by a painful and prolonged erection of more than four hours in the absence of sexual stimulation. Ischaemic priapism has an incidence of 5.34 per 100,000 males and is the most common presentation of priapism, accounting for 95% of cases [1, 2]. Several aetiological factors contribute to the development of ischaemic priapism, and over the past few years, pharmacological causes have been gaining attention. Today, drug-induced ischaemic priapism is the most common [3, 4], and the different aetiologies will be explored. Erectile dysfunction (ED) is a critical sequelae of ischaemic priapism. A decrease in smooth muscle cells and an increase in tissue fibrosis occur during ischaemic priapism [5]. These changes are time-sensitive and directly correlate with the duration of the ischaemic episode [5]. It is invaluable for medical practitioners to be aware of the various drugs that can induce possible ischaemic priapism and how to manage it promptly.

## Methods and materials

This narrative review was conducted on the 11^th^ of June 2023 to assess all available literature on pharmacologically induced ischaemic priapism up to the present day. The search was performed using the PubMed database (US National Library of Medicine, Bethesda, Maryland) and Google Scholar. Papers were analysed using the following combination of MeSH terms: (“pharmacological aetiology” OR “drug-induced” OR “medication-induced”) AND ischaemic priapism, resulting in 210 original papers published. After reviewing each article, 95 articles were deemed relevant and in keeping with the objective. Bibliographies of essential articles were also studied to find papers of additional interest.

### Ischaemic priapism

Ischaemic priapism (veno-occlusive, low flow) is persistent erection (>4 h) characterised by little or no cavernous venous outflow, which hinders arterial inflow and subsequently leads to smooth muscle and endothelial damage, tissue ischaemia, and eventually fibrosis [6]. The patient typically complains of penile pain, and examination reveals a rigid erection [7].

### Common pharmacological aetiology of ischaemic priapism

The timely diagnosis and treatment of ischemic priapism have a direct effect on long-term erectile function. Thus, the attending physician must understand the mechanism and possible aetiology. Of the many aetiologies of ischaemic priapism, drug-induced ischaemic priapism is now the most common cause of ischaemic priapism [3, 4]. Drug-induced ischaemic priapism is commonly associated with antidepressants, antipsychotics and antihypertensive medications, which account for approximately 15–41% of all cases, of which antipsychotic-induced ischaemic priapism is most common, accounting for 50% of drug-induced instances [8, 9]. Of significant concern, data published by the FDA conducting the most extensive review of drug-induced ischaemic priapism found that 55.7% were 40 years old or younger with a median age of 36 [4]. The following is a comprehensive review of the common pharmacological causes of ischaemic priapism, with an extensive list of documented causes in Table 1.

**Table 1:** Pharmacological aetiology of ischaemic priapism.

Drug class	Subtype	Examples	Route of administration	Proposed mechanism of action	Incidence of ischaemic priapism
Antipsychotics	Typical	Phenothiazine (Chlorpromazine, Fluphenazine, Thioridazine, Prothipendyl hydrochloride)	IM/IV/Oral	*α*-adrenergic blockade	Account for approximately 50% of pharmacologically induced ischaemic priapism [12].
		Haloperidol, Zuclopenthixol, Pipamperone	IM/IV/Oral		
	Atypical	Clozapine, Risperidone	IM/IV/Oral		
		Olanzapine, Aripiprazole, Quetiapine, Paliperidone, Iloperidone, Lithium, Ziprasidone			
Anti- depressants	Serotonin receptor antagonists and reuptake inhibitors (SARI)	Trazodone	Oral	*α*-adrenergic blockade	Trazodone accounts for 16% of pharmacologically induced ischaemic priapism [4].
	Selective serotonin reuptake inhibitors (SSRI)	Fluoxetine, Citalopram, Paroxetine	Oral		
	Norepinephrine and dopamine reuptake inhibitor (NDRI)	Bupropion	Oral		
	Selective serotonin and norepinephrine reuptake inhibitors (SNRIs)	Venlafaxine, Duloxetine, Atomoxetine	Oral		
Alpha-adrenergic receptor antagonists	Selective	Tamsulosin, Alfuzosin, Moxonidine, Terazosin, Silodosin	Oral	*α*-adrenergic blockade	20 reported cases [19–21, 58, 59].
	Non-Selective	Doxazosin, Prazosin, Terazosin, Phentolamine	Oral		
Central nervous system stimulant		Methylphenidate	Oral	Increased dopamine release	10 reported cases [60–69].
Vasoactive erectile agents	Phosphodiesterase 5 (PDE5) inhibitor	Sildenafil, Tadalafil	Oral	Increase synthesis of nitric oxide (NO).	9% of published drug-induced priapism cases [4]. Only 42.5% of these cases were PDEI5s taken appropriately and not in association with concomitant drugs [4].
	Benzylisoquinoline alkaloid	Papaverine hydrochloride	Oral/Intracavernous injection	Increased smooth muscle relaxation of the corpora cavernosa. Increased resistance of venous outflow.	Account for 2.68% of intracavernous injections with papaverine hydrochloride [29].
	Prostaglandin E1	Alprostadil	Oral/Intracavernous injection/Topical/ Intraurethral	Increase smooth muscle relaxation and vasodilation.	1% of intracavernous injections with alprostadil [70].
Anaesthetics	Opioid	Morphine	Epidural	Undefined	3 reported cases [71–73].
	Non-barbiturates	Propofol	IV	Overstimulation of the parasympathetic system. Increased synthesis of NO.	5 reported cases [74–78].
Hormonal therapy	Testosterone	Testosterone enanthate	IM	Upregulation of NO synthase.	22 reported cases [79–93].
	Gonadotropin releasing hormone		IM		2 reported cases [94].
	Androstenedione		Oral		1 reported case [95].
	Cabergoline + Bromocriptine		Oral	*α*-adrenergic receptor antagonism and dopaminergic receptor agonism.	3 reported cases [96–98].
Anticoagulants	Heparin	Unfractionated heparin Low molecular weight heparin	IV/IM	Platelet aggregation leading to veno-occlusion triggered by antiplatelet antibodies.	At least 35 reported cases, mostly associated with unfractionated heparin [99–110].
	Coumarins	Warfarin	Oral	Undefined	Unknown [111].
		Acenocoumarol	Oral	Undefined	1 reported case [112].
Chemotherapy	Alkylating agent	Oxaliplatin	IV	Undefined	1 reported case [56].
Dopamine agonist	Non-ergoline	Rotigotine	Transdermal patch	Undefined	1 reported case [113].
Over the counter medication	Tribulus terrestris		Oral	Undefined	1 reported case [114].
	Rhino 7 Platinum 3000		Oral	Undefined	1 reported case [115].
Anti-Convulsant	Second generation antiepileptic	Topiramate	Oral	Undefined	1 reported case [116].
	First generation anti-epileptic	Sodium valproate	Oral	Undefined	1 reported case [117].
	Gabapentinoids	Pregabalin	Oral	Undefined	2 reported cases [118, 119].
		Gabapentin	Oral	Undefined	1 reported case [120].
	Phenyltriazine	Lamotrigine	Oral	Undefined	1 reported case [121].
Monoclonal antibody	Tumour necrosis factor blocker	Adalimumab	Subcutaneous	Excess local NO production.	1 reported case [122].
Antibiotics	Glycopeptides	Vancomycin	IV	Undefined	2 reported cases [123, 124].
Melanocortin	Melanotan II		Subcutaneous	Undefined	3 reported cases [125, 126].
Parenteral nutrition	20% fat emulsion		IV	Increase in blood coagulability, and fat embolism.	Unknown [127–129].
Illicit drugs	Psychoactive drugs	Cocaine	Oral/Intracavernous injection/Topical/Intranasal	Sympathetic neurotransmitter depletion.	Unknown [32, 34, 130, 131].
		Marijuana	Oral/inhaled	Increased parasympathetic activity which is cannabinoid-mediated.	1 reported case, excluding concurrent drug use [132].
		Methylenedioxymethamphetamine	Oral	Dopamine agonism and serotonin depletion.	Unknown [33, 36].

### Antipsychotics

The exact mechanism of ischaemic priapism associated with antipsychotic medications remains unknown, but the commonly proposed mechanism is an alpha-adrenergic blockade in the corpora cavernosa of the penis [10]. Specifically, alpha-1-receptor blockade leads to direct arteriolar dilatation, which results in an increase of blood inflow and a decreased outflow secondary to emissary veins obstruction [11]. Antipsychotic medications are broadly classified as typical and atypical antipsychotics, as seen in Table 1. Both classes are implicated in cases of ischaemic priapism regardless of the alpha-1 adrenergic receptor affinity [4]. Typical antipsychotics most commonly involved are phenothiazines(fluphenazine, chlorpromazine, thioridazine, prothipendyl hydrochloride) and haloperidol [4, 12]. Atypical antipsychotics involved are risperidone, clozapine, quetiapine, olanzapine, aripiprazole and ziprasidone [12]. The FDA showed that 4 of the top 5 medications associated with ischaemic priapism are quetiapine, risperidone, olanzapine and aripiprazole, which are all atypical antipsychotics [4]. Risks of developing ischaemic priapism on antipsychotic medication include increasing the dose, switching to different classes of antipsychotics, and restarting treatment after abstinence [4]. The prevalence of prescribed and “off-label” use of antipsychotic medications has been on the rise in recent years [13]. Therefore, medical practitioners in all disciplines should know that early intervention and appropriate treatment are essential to prevent permanent ED and fibrosis [14].

### Antidepressants

With the global push for Mental Health awareness and improved diagnostics, antidepressant medications are well documented in pharmacologically induced ischaemic priapism, with varying proposed mechanisms depending on the class of drugs. With a 48% increase in worldwide depression cases between 1970 and 2017, there is an elevated risk of ischaemic priapism for patients [15]. Documented medications are mainly classed into serotonin receptor antagonists and reuptake inhibitors (SARI), selective serotonin reuptake inhibitors (SSRI), norepinephrine and dopamine reuptake inhibitors (NDRI) and selective serotonin and norepinephrine reuptake inhibitors (SNRIs). Many research papers have been published, and it is widely accepted that antidepressant medications cause ischaemic priapism by their alpha-1-adrenergic antagonism [16], decrease in local sympathetic tone [17] and increase in peripheral serotonin levels through re-uptake inhibition [18]. One drug that falls in this category with extensive supporting literature is trazodone, which has the highest associated risk with ischaemic priapism of all drugs. Trazadone is classified as a SARI and alone accounts for 15.98% of medication-induced ischaemic priapism between the years 2015 and 2020 [4]. Trazadone is proposed to have alpha-adrenergic blockade properties, and caution should arise when prescribing it in patients with concomitant risk factors for ischaemic priapism.

### Alpha adrenoreceptor antagonist

Table 1 shows alpha-blockers have different selectivity profiles, each with benefits for the specific use case. It is broadly differentiated into selective (block alpha-1 receptors) and non-selective (block both alpha-1 and -2 receptors). Due to the difference in alpha receptor selectivity and the dosage required, alpha-blocker-induced ischaemic priapism is more frequently documented in patients being treated with hypertension [19]. However, monotherapy with recommended doses and the risk of ischaemic priapism with these agents has not been widely investigated but is documented in case reports [20–24].

### Vasoactive erectile agents

Oral phosphodiesterase five inhibitors (PDE5I) prescribed alone in a medical setting are unlikely to cause ischaemic priapism, with only a few case reports noted. This is in contrast with an international cohort study, which showed a significant increase in the incidence of priapism with the use of PDE5Is [25]. Still, it could include inappropriate use and confounding conditions, which must be investigated [25]. A significant amount (57.5%) of the reports associated with ischaemic priapism were related to concomitant PDE5I and illicit drugs taken at the same time and/or inappropriate intake/excessive dosage [4].

### Intracavernosal injection (ICI)

Ischaemic priapism occurs at an incidence of 0.25–7.3% after ICI for the treatment of ED [26–28]. There is a higher risk of ischaemic priapism with papaverine combinations than prostaglandin E1 injections [14, 29]. A recent cohort on adverse drug reaction events of erectogenic medication showed ischaemic priapism occurred 25 times more from ICI than PDE5I [30]. A cross-sectional analysis which assessed the risk of priapism after ICI revealed that patients who did present with priapism were likely to be younger, had sickle cell disease, and had a higher prevalence of mood or behavioural disorders [31].

### Recreational drugs and substances

Through extensive review, cocaine, mainly in chronic use, and marijuana in combination with methylenedioxymethamphetamine (MDMA) have been documented in case reports, however rarely, to be a cause of ischaemic priapism [32, 33]. It is accepted that cocaine can inhibit the re-uptake of norepinephrine by blocking transport in the presynaptic sympathetic neuron, which results in an abundance of norepinephrine in the synaptic cleft and the consequent propagation of sympathetic discharge [34]. With the use of MDMA, however, it is hypothesised that the drug affects both serotonin and dopamine pathways [35–38]. It is imperative that a specific questioning of drug use is obtained or toxicology ordered if there is adequate clinical suspicion. Recreational use of PDE5I and ICI has also been implicated and is an essential cause of ischaemic priapism in men who do not have ED. A retrospective review in a Los Angeles tertiary medical centre found recreational ICIs accounted for 49% of all ischaemic priapism treated in that centre [39]. Due to inadequate information published globally, the true risk cannot be assessed. Therefore, improved data capturing on a local and regional scale is advised.

### Other

Multiple hypothesised mechanisms, both known and unknown to the medical field, can be affected by drugs and are implicated in the pathogenesis of ischaemic priapism. Table 1 refers to all possible aetiologies of pharmacologically induced ischaemic priapism found after extensive PubMed and Google Scholar research using the abovementioned methods.

### Diagnostic evaluation of suspected drug-induced priapism

A thorough and precise history and examination are essential for effective time management in the treatment of ischaemic priapism, which can lead to decreased morbidity. A focused examination will assist in establishing the presence of pain, trauma and underlying malignancy. The glans penis is usually soft, while the corpora are tender and fully rigid. Upon history taking, the following should be emphasised [2]:

- Duration of erection
- Association and severity of pain
- Episodes and prior treatment of ischaemic priapism
- Current erectile function
- Medication and drug use
- Medical history: Sickle cell disease, haemoglobinopathies, hypercoagulable states, vessel vasculitis
- Trauma or surgery to the pelvis, perineum, or penis

Penile MRI likely does not have a role in the initial diagnostic and treatment phase of ischaemic priapism in acute settings due to the time-sensitivity of ischaemic priapism [40]. However, in the cases of refractory ischaemic priapism or delayed presentation (>48 h), smooth muscle viability can be indirectly assessed [40]. In a prospective study of 38 patients with ischaemic priapism, the sensitivity of MRI in predicting non-viable smooth muscle was 100% when correlated with corpus cavernosum biopsies [40].

### Assessment and further workup

**Fig. 1:**
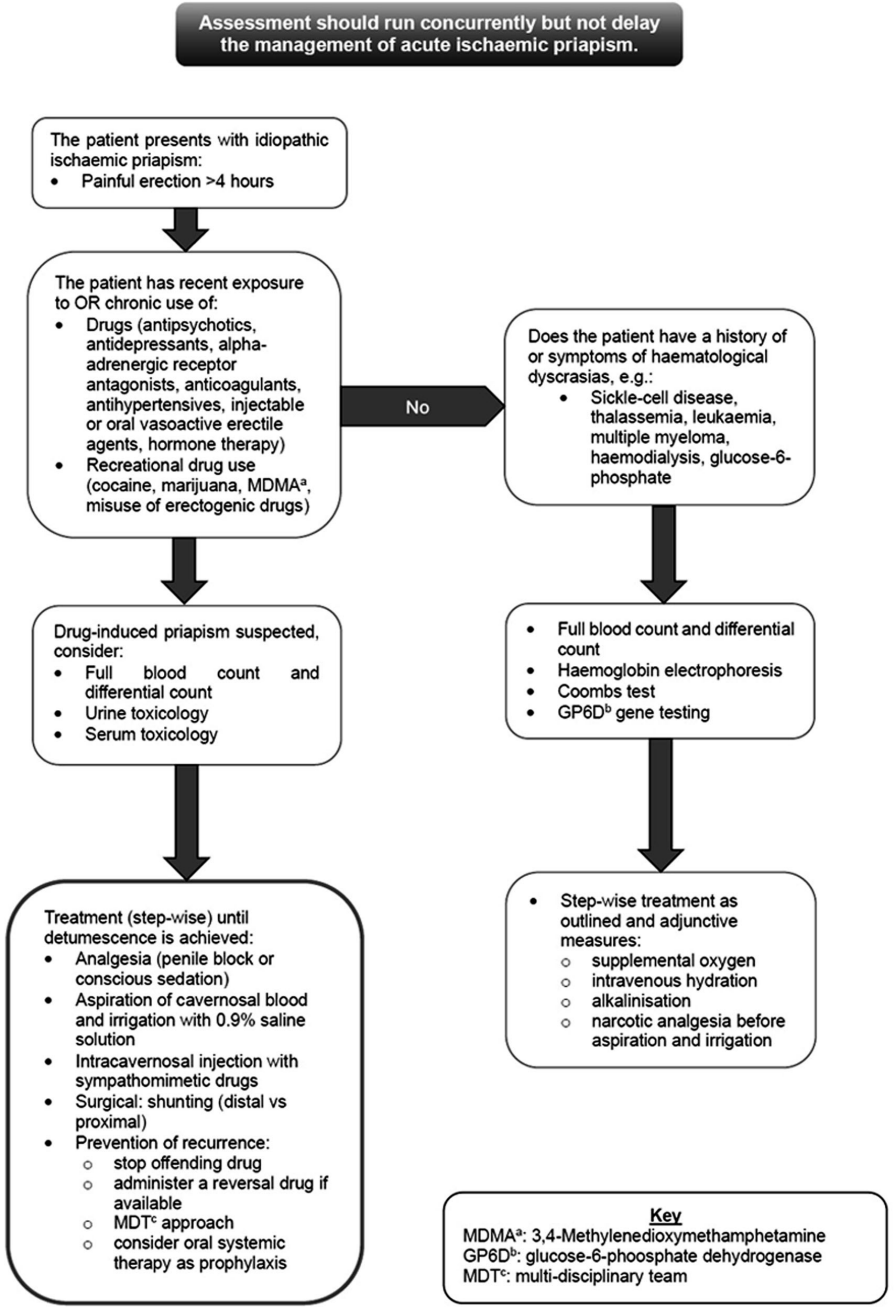
Proposed assessment and management algorithm for idiopathic ischaemic priapism.

Based on the current understanding of pharmacologically induced ischaemic priapism and improved reporting of aetiologies, we propose an algorithm in Fig. 1 to be used by clinicians to assist with assessing possible causes of idiopathic ischaemic priapism. It is important to note that the workup of patients with ischaemic priapism should run concurrently and not delay acute management. As of recent years, pharmacological causes are currently the most prevalent; however, haematological dyscrasias, specifically sickle cell disease, have an incidence as high as 35% [41]. The assessment approach proposed streamlines the multiple aetiologies by addressing the most prevalent first.

### Management of suspected drug-induced priapism

For desirable outcomes, first-line treatment should be initiated within 24 h of presentation [42]. The aim is to restore detumescence in the absence of pain to prevent ED and subsequent corporal fibrosis. ED severity is directly correlated to the duration of the ischaemic priapism with rates of up to 100% of ED in ischaemic priapism over 48 hr duration, 50–60% with 36–48 h duration, and 0–20% in less than 24 h duration [42].

Analgesia before intracavernosal blood aspiration and irrigation can be achieved by administering a block or conscious sedation. The first-line intervention involves corporal blood aspiration and irrigation with 0.9% saline solution [43]. Access into the corpora can be achieved through the glans or to the lateral aspect of the penile shaft. Aspiration and irrigation can be combined with the intracavernosal injection of sympathomimetic drugs [44]. If first-line treatment fails, there is delayed presentation of ischaemic priapism, or in refractory cases, second-line intervention can be considered [45]. This refers to surgical intervention in the form of shunting and immediate penile prosthesis implantation [46].

Further treatment would include discontinuation of the offending drug and replacing it with a different class that has a lower risk of iatrogenic ischaemic priapism. If a drug reversal is present for the offending agent, it should be considered if available.

In the case of recurrent ischaemic priapism, acute episodes should be treated the same as ischaemic priapism. There is limited evidence to support oral systemic drugs (α-adrenergic agonists, dutasteride, digoxin, gabapentin, baclofen, and hydroxyurea) and should be initiated with caution to their individual side effects [47–49]. There is reported success with hormonal therapies such as ketoconazole and cyproterone acetate for the treatment of recurrent ischaemic priapism. Still, it has associated toxicity and side effects when manipulating the hypothalamic-pituitary-gonadal axis, including hot flashes, mood disorders, ED, fatigue, and breast tenderness [48]. PDE5Is at low doses have been shown to exert a paradoxical effect in preventing and alleviating recurrent ischaemic priapism [50].

To prevent recurrence and subsequent complications, a multidisciplinary team (including a pharmacist, psychiatrist, toxicologist, urologist, and paediatrician) is of the utmost importance.

## Discussion

ED has been shown to have a direct correlation with decreased self-esteem, confidence, and relationship satisfaction and can have adverse effects on long-term psychosocial quality of life [51]; therefore, timeous intervention should be prioritised when addressing patients with ischaemic priapism. With evidence showing a greater prevalence in the age group below 40, understanding the everchanging scope of drug-induced ischaemic priapism has the potential to improve long-term morbidity in a urological setting. Due to mental illnesses and the increased use of antipsychotic medication [52], antidepressants [53] and recreational drug use [54] it stands to reason to expect an increase in the incidence of ischaemic priapism in the general population. Specific attention towards the use of antipsychotics is relevant as it is responsible for approximately 50% of all drug-induced ischaemic priapism [12]. It is of concern that there has already been a proven increase in incidence in the paediatric population due to antipsychotics [55]. With the proposed assessment algorithm, we aim to establish a standardised method to pinpoint potential causes. Consequently, this can lower the recurrence rate and enhance the timeliness of interventions. Patient awareness of pharmacological side effects should be encouraged by all clinical practitioners as this will improve health-seeking behaviour and decrease the time needed for intervention.

Two significant limitations noted when performing this narrative review that could be addressed in future medical research are drug-drug and drug-disease interactions, as seen in a case report reviewing a patient undergoing first-line chemotherapy for metastatic colon cancer, who subsequently sustained an ischaemic priapism post oxaliplatin infusion [56]. With no known information on the mechanism of action or subsequent reporting on other occurrences, it does pose a medical challenge to identify all possible physiological changes due to medical conditions on an individualised basis. Drug-drug interactions can also have a varied and unpredictable effect on physiology. With a known effect of inhibiting cytochrome P450 (CYP450) enzyme, it is hypothesised that the antiretroviral regime in HIV patients was likely the primary culprit in causing greater than expected free levels of perphenazine and quetiapine resulting in ischaemic priapism [57]. It has also been postulated that there are possible signals for ischaemic priapism in patients taking antiseizure medication, however further studies are needed to better define this [10]. The endeavour of conducting medical research, particularly in the realm of pharmacology, is fraught with complexities stemming from drug-drug interactions and drug-disease interactions. These complex interactions between drugs and the human body present significant research challenges. Drug-drug interactions can lead to unexpected synergistic or antagonistic effects when multiple medications are administered concurrently, necessitating comprehensive investigations to ensure patient safety. Moreover, the complexities introduced by drug-disease interactions further compound the research difficulty, as underlying medical conditions can alter drug metabolism, efficacy, and safety profiles. Such multifaceted interactions underscore the critical need for meticulous study design, comprehensive data analysis, and ongoing medical research to better understand and mitigate the potential side effects and risks associated with pharmacotherapy.

## Conclusion

Ischaemic priapism, though characterised by a relatively low incidence rate, looms large with its substantial morbidity burden. Elevating our comprehension and vigilance regarding pharmacology among patients and across medical specialities is necessary to pursue enhanced long-term patient well-being. Drug-induced ischaemic priapism is the most common cause of ischaemic priapism, and its clinical significance is underestimated, considering the widespread use of these implicated medications. In this context, the medical community needs to emphasise the critical importance of managing this condition with precision and diligence, safeguarding patient health, and advancing the frontiers of urological care. This can be done with input from a multidisciplinary team, including pharmacists, toxicology, psychiatry, and other specialities like paediatrics.
